# A Manual of the Practice of Medicine

**Published:** 1900-05

**Authors:** 


A MANUAL OF THE PRACTICE OF MEDICINE. Prepared Especially for Students. By A. A. STEVENS, A. M., M. D., Professor of Pathology in the Woman's Medical College of Pennsylvania; Lecturer on Terminology and Instructor in Physical Diagnosis in the University of Pennsylvania; Physician to St. Agnes's Hospital and Out-patient Department of the Episcopal Hospital, etc. Fifth edition, revised and enlarged. Illustrated. Philadelphia: W. B. Saunders. 1900.

This excellent manual of the practice of medicine is designed especially for the use of students. This edition has been thoroughly revised. The chapters on diseases of the pancreas and on diseases of the blood and of the ductless glands, as well as the articles on appendicitis, angina pectoris, aphasia, myxedema, and syringomyelia, have been entirely rewritten. Other quite new articles have been added. The fact that this is the fifth edition is of itself sufficient comment on the value of the work. The book is a handsome one, with red edges and soft leather covers. M. J. F.

